# A recombinant IgG1 Fc-domain protein ameliorates inflammatory demyelinating peripheral neuropathy

**DOI:** 10.3389/fimmu.2026.1857016

**Published:** 2026-05-27

**Authors:** Husniye G. Otlu, Tong Gao, Greg P. Coffey, Jessica M. Bright, Alyssa M. A. Toda, Pamela B. Conley, Kazim Sheikh, Gang Zhang

**Affiliations:** 1 Neuromuscular Research Laboratory, Department of Neurology, McGovern Medical School, The University of Texas Health Science Center at Houston, Houston, TX, United States; 2 Vocational Health Sciences, Laboratory Techniques Program, Malatya Turgut Ozal University, Malatya, Türkiye; 3 Nuvig Therapeutics, Menlo Park, CA, United States

**Keywords:** chronic inflammatory demyelinating polyneuropathy, FcγRIIB, Guillain-Barré syndrome, intravenous immunoglobulin, NVG-2089, Treg, type II Fc receptors

## Abstract

**Introduction:**

Guillain–Barré syndrome (GBS) and chronic inflammatory demyelinating polyneuropathy (CIDP) are immune-mediated inflammatory neuropathies characterized by T cell– and/or autoantibody-driven injury to peripheral nerves. Although intravenous immunoglobulin (IVIg) is effective in both conditions, its use is limited by high dosing requirements, supply constraints, and manufacturing complexity. NVG-2089 is a recombinant IgG1 Fc-domain protein engineered to selectively agonize type II Fc receptors, promoting anti-inflammatory signaling through expansion of regulatory T cells (Tregs) and upregulation of the inhibitory Fcγ receptor FcγRIIB. Its efficacy in immune complex–mediated disease and T cell–driven neuroinflammation prompted evaluation of its therapeutic potential in an animal model of inflammatory demyelinating neuropathy.

**Methods:**

We conducted a randomized, blinded preclinical study in the spontaneous autoimmune peripheral polyneuropathy model, a well-established murine model of inflammatory demyelinating neuropathy. Animals were treated with NVG-2089, high-dose IVIg, or vehicle. Outcomes included behavioral performance, nerve electrophysiology, peripheral nerve morphometrics, immunohistochemistry, and flow cytometric immunophenotyping.

**Results:**

Both NVG-2089 and IVIg stabilized motor and sensory performance, preserved compound muscle action potential (CMAP) amplitudes, and attenuated demyelination-associated prolongation of distal latency and CMAP duration. Morphometric analyses demonstrated preservation of myelinated fiber density and normalization of g-ratio distributions in both treatment groups. Immunophenotyping indicated an expansion of functionally activated CD25^+^CD39^+^ Tregs in the spleen, along with increased FcγRIIB expression on B cells, monocytes, and dendritic cells across systemic and nerve-associated compartments, consistent with engagement of shared anti-inflammatory pathways.

**Conclusions:**

NVG-2089 demonstrated robust neuroprotective efficacy comparable to IVIg while requiring a substantially lower protein dose. These findings support NVG-2089 as a promising, dose-efficient alternative to IVIg for the treatment of inflammatory demyelinating neuropathies, including GBS and CIDP.

## Introduction

Guillain–Barré syndrome (GBS) and chronic inflammatory demyelinating polyneuropathy (CIDP) represent the most common acute and chronic immune-mediated inflammatory neuropathies encountered in clinical practice, respectively. GBS is an acute polyradiculoneuropathy and the leading cause of acute flaccid paralysis worldwide (1, 2), characterized by rapidly progressive weakness, areflexia, and variable sensory and autonomic involvement. It comprises two major subtypes: demyelinating and axonal. The predominant form in western populations is acute inflammatory demyelinating polyneuropathy (AIDP), mediated by T cell– and macrophage-driven immune injury targeting peripheral nerve myelin (3, 4), whereas axonal variants are less frequent in these regions but more prevalent in developing countries and parts of Asia, including Japan (5–8). This study focuses on the demyelinating spectrum of inflammatory neuropathies, specifically AIDP and CIDP, given their shared immunopathogenic mechanisms. CIDP represents the chronic counterpart, with a progressive or relapsing–remitting course over ≥8 weeks, characterized by symmetric weakness, sensory impairment, and electrophysiological evidence of demyelination. Both AIDP and CIDP involve heterogeneous immune mechanisms, including cellular and humoral responses directed against peripheral nerve components (1, 9, 10). The annual incidence of GBS is estimated at approximately 1–2 cases per 100,000 individuals, with higher risk in males and older adults (11, 12), while CIDP occurs less frequently but contributes substantially to long-term neurological disability. Epidemiologic studies consistently demonstrate a male predominance in GBS, with reported male-to-female ratios commonly around 1.5:1, while CIDP also shows male predominance, with ratios often ranging from approximately 1.5:1 to 2:1 depending on the cohort and diagnostic criteria used (11, 13–16). The mechanisms underlying this sex difference remain incompletely defined but are likely multifactorial, potentially involving sex-related differences in immune regulation, hormonal and genetic influences, unconventional T-cell responses, age-related immunosenescence, and possible differences in peripheral nerve susceptibility to immune-mediated injury (17, 18). Despite advances in supportive care and immunotherapy, both disorders are associated with substantial morbidity, including incomplete recovery, persistent weakness, sensory deficits, neuropathic pain, fatigue, and treatment-related complications (19).

Intravenous immunoglobulin (IVIg) is accepted as a first-line therapy for both GBS and CIDP. However, widespread reliance on IVIg is increasingly constrained by several practical and systemic limitations. IVIg is costly and derived from a complex, plasma-based manufacturing process that requires pooled polyclonal IgG collected from thousands of healthy donors. This dependence on donor plasma introduces fragility into the supply chain, rendering availability vulnerable to fluctuations in donor participation and manufacturing capacity. Standard dosing for GBS and CIDP, typically 2 g/kg administered over several days, further amplifies demand on global IVIg supplies, contributing to recurrent shortages and placing significant strain on healthcare systems worldwide (20–22). In addition to supply constraints, IVIg administration requires prolonged intravenous infusions and is associated with infrequent but clinically meaningful adverse events, including headache, aseptic meningitis, renal dysfunction, and thromboembolic complications (23–25). Collectively, these limitations reinforce the need for more dose-efficient, scalable alternatives that preserve the therapeutic efficacy of IVIg while improving access, tolerability, and long-term sustainability.

Mechanistically, IVIg is thought to exert broad immunomodulatory effects through multiple, partially overlapping pathways. These include modulation of activating and inhibitory Fcγ receptor signaling, most notably the upregulation and engagement of the inhibitory Fcγ receptor FcγRIIB, partial saturation of the neonatal Fc receptor (FcRn) leading to accelerated clearance of pathogenic IgG, inhibition of complement activation, neutralization of autoantibodies and cytokines, and expansion of regulatory T cell (Treg) populations (26–28). A growing body of evidence suggests that a substantial component of IVIg’s anti-inflammatory activity can be attributed to a minor fraction of IgG molecules bearing α2,6-linked sialylation on the Fc glycan (29, 30). This sialylated Fc subset is thought to engage type II Fc receptors, such as SIGN-R1 in mice and DC-SIGN in humans, thereby initiating regulatory signaling cascades that suppress inflammation and promote immune tolerance, although aspects of receptor specificity and signaling in humans remain an area of active investigation (21, 30, 31). These mechanistic insights have catalyzed interest in rationally engineered therapies designed to selectively recapitulate the beneficial immunomodulatory effects of IVIg without the need for high-dose, plasma-derived polyclonal IgG.

Inhibitory Fcγ receptors, particularly FcγRIIB (CD32B), play a central role in maintaining immune homeostasis by counterbalancing activating Fcγ receptor signals. Engagement of FcγRIIB leads to recruitment of the phosphatases SHIP-1 and SHP-1 via its immunoreceptor tyrosine-based inhibition motif (ITIM), resulting in attenuation of B-cell receptor and Fc receptor–mediated signaling pathways (32). Through these mechanisms, FcγRIIB limits cytokine production, restrains myeloid cell activation, and enforces tolerance across both B-cell and innate immune compartments (33, 34). Genetic deletion or functional impairment of FcγRIIB exacerbates autoimmune disease phenotypes in multiple experimental systems and accelerates demyelinating pathology within peripheral nerves (35). In contrast, enforced or selective activation of FcγRIIB signaling, achieved through engineered IgG Fc domains, has been shown to reproduce key protective effects of IVIg, including restoration of immune regulatory networks and expansion of IL-10–producing B cells and Tregs (31, 36). These findings support FcγRIIB as a compelling therapeutic target for antibody- and T cell–mediated neuropathies such as GBS and CIDP, with the potential to overcome the dosing inefficiencies and supply constraints inherent to conventional IVIg therapy.

Building on this mechanistic framework, engineered Fc-domain–based therapeutics have been developed to preferentially adopt sialylated-Fc–like conformations and selectively activate type II Fc receptor pathways, thereby promoting immune regulation without inducing broad immunosuppression. NVG-2089 is an engineered human IgG1 Fc variant containing a F241A substitution that confers a sialylated Fc-like conformation and selectively activates type II Fcγ receptor signaling (21). Through this mechanism, NVG-2089 promotes Treg expansion and suppresses pathogenic inflammatory responses (31). In preclinical studies, NVG-2089 has demonstrated robust efficacy across a range of immune complex– and T cell–driven disease models, including experimental autoimmune encephalomyelitis and inflammatory arthritis, highlighting its potential as a mechanistically defined, dose-efficient alternative to IVIg (21). In principle, an Fc-domain–only therapy offers several advantages over plasma-derived IVIg, including improved scalability, reduced protein load, and more precise engagement of immunoregulatory pathways.

Rigorous preclinical evaluation is essential to determine whether engineered Fc-domain therapies can replicate the clinically meaningful benefits of IVIg in relevant disease contexts. The spontaneous autoimmune peripheral polyneuropathy (SAPP) model observed in non-obese diabetic mice deficient in the costimulatory molecule B7-2 (NOD.B7-2^-^/^-^) represents a well-established model of spontaneous progressive inflammatory demyelinating neuropathy (37). Because this study focuses on the demyelinating spectrum of inflammatory neuropathies, the SAPP model provides a biologically relevant platform for evaluating therapeutic effects. The disease in this model is driven by T cell– and macrophage-mediated immune responses and is characterized by endoneurial inflammation, segmental demyelination, and secondary axonal degeneration (38). Importantly, SAPP recapitulates key histopathological and immunological features of human inflammatory demyelinating neuropathies, supporting its use as a robust system for assessing immunomodulatory interventions (39, 40).

In this study, we assessed the therapeutic efficacy of NVG-2089 in comparison with high-dose IVIg in the SAPP model. We employed a prespecified, multi-level outcome framework encompassing behavioral assessments, nerve conduction electrophysiology, quantitative peripheral nerve morphometrics, and flow cytometric immunophenotyping of both systemic and local immune cell populations. We hypothesized that NVG-2089 would confer neuroprotection and immunomodulatory effects comparable to those of IVIg while requiring a substantially lower total protein dose, consistent with selective activation of shared sialylated Fc–type II Fc receptor and FcγRIIB-dependent regulatory pathways.

## Materials and methods

### Animals and experimental design

Mice were housed under a 12-h light/dark cycle with ad libitum access to food and water. All experimental procedures were conducted in accordance with institutional and governmental guidelines and were approved by the UTHealth Institutional Animal Care and Use Committee (AWC-23-0034). Only female B7–2 knockout non-obese diabetic (NOD) mice were used in this study because this model exhibits high disease penetrance in females.

**Figure 1:**
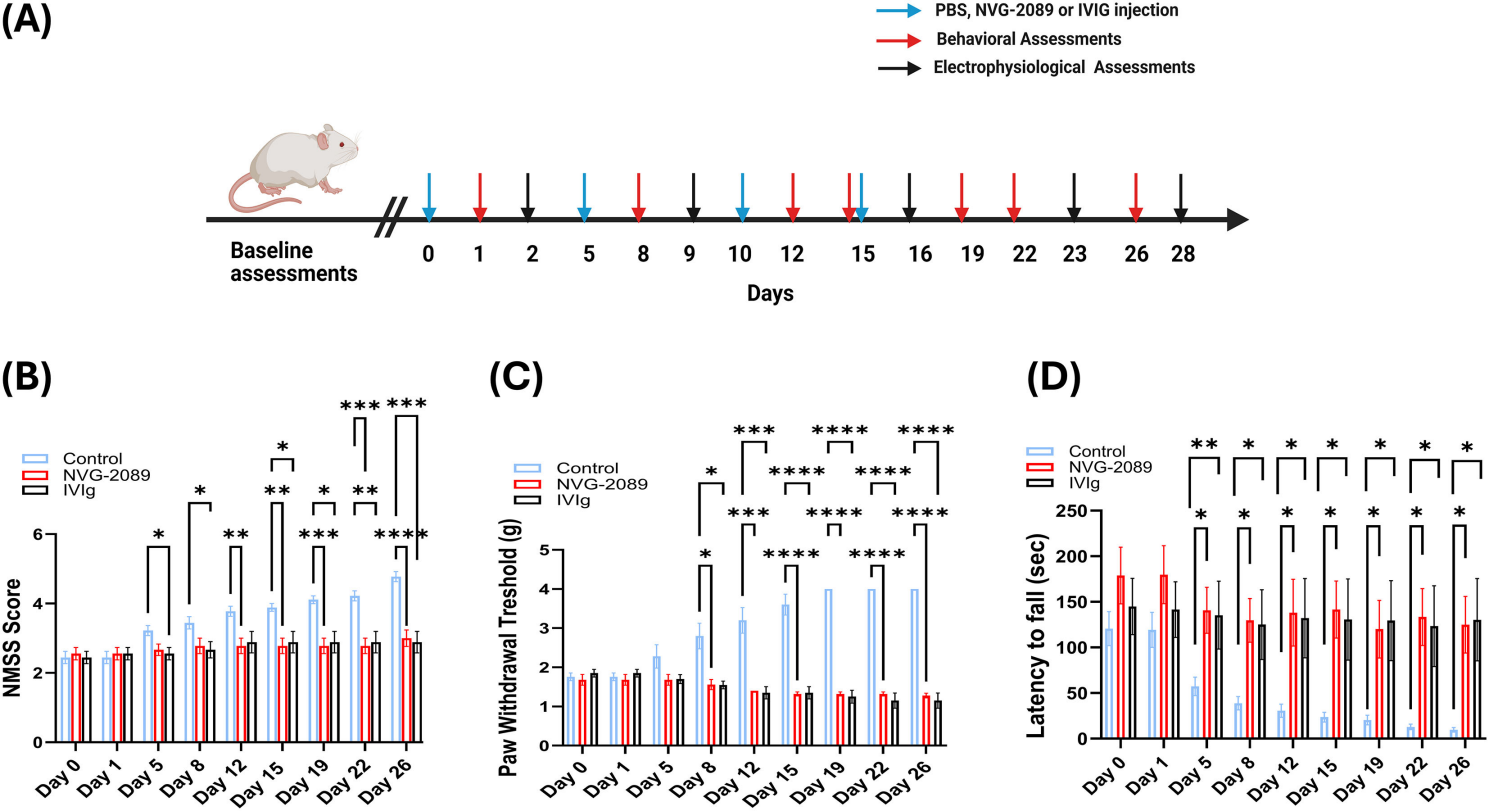
Experimental design and functional outcomes following NVG-2089 or IVIg treatment. **(A)** Schematic overview of the study timeline, including baseline assessments, treatment administration (PBS, NVG-2089, or IVIg; blue arrows), and longitudinal behavioral (red arrows) and electrophysiological (black arrows) evaluations over 28 days. **(B)** Neuromuscular Severity Score (NMSS) assessed at baseline and throughout follow-up. Both NVG-2089– and IVIg-treated groups demonstrated significantly lower NMSS values compared with controls, indicating attenuation of disease progression. **(C)** Paw withdrawal threshold testing revealed reduced mechanical sensitivity in control animals, whereas NVG-2089 and IVIg significantly preserved sensory function. **(D)** Rotarod performance (latency to fall) showed sustained motor coordination in NVG-2089– and IVIg-treated animals compared with controls. Data are presented as mean ± SEM with individual data points shown. n=8–10 mice per group. *p < 0.05, **p < 0.01, ***p < 0.001, ****p < 0.0001; ns, not significant.

Prior to treatment, animals underwent baseline electrophysiological and behavioral assessments and were randomly assigned to one of three treatment groups: phosphate-buffered saline (PBS) control (volume matched to NVG-2089, i.p.); IVIg (1 g/kg, i.p.); or NVG-2089 (100 mg/kg, i.p.). Treatments were administered every 5 days over a 28-day period. Behavioral and electrophysiological outcomes were assessed longitudinally to evaluate recovery. A schematic of the experimental design is shown in **Figure 1A**.

The study was performed using two independent cohorts. The longitudinal phenotypic assessment cohort included 8–10 female mice per treatment group. The flow cytometric immunophenotyping cohort included 8 female mice per treatment group. Thus, each experiment included female mice only, with group sizes selected before study initiation.

Sample sizes were determined based on multiple factors, including prior published studies using similar experimental designs, preliminary findings from our own investigations, and formal power calculations performed in consultation with a statistician. Power analyses were conducted to ensure that the studies were sufficiently powered to detect biologically meaningful differences among treatment groups while avoiding the use of an excessive number of animals.

### Spontaneous autoimmune peripheral polyneuropathy animal model

The SAPP model was developed in NOD mice deficient in the costimulatory molecule B7-2 (NOD-B7.2^-/-^) and pathologically mimics CIDP and the demyelinating form of GBS (37, 41). Auto-reactive T cells directed against myelin protein zero are critical in SAPP pathogenesis as adoptive transfer of these cells can produce inflammatory demyelination (42). For several reasons, this is considered the most representative model to study T cell–mediated inflammatory demyelinating nerve injury. We and others have previously characterized the temporal clinical and pathological features of neuropathy in these animals (35, 43). Mice were bred and maintained in our institutional animal facility. Disease severity was assessed using the Neuromuscular Severity Score (NMSS), an ordinal scale ranging from 0 to 5 that quantifies muscle weakness based on established criteria, with higher scores indicating greater disease severity (35, 44). Only NOD-B7.2^-/-^ mice exhibiting a moderate disease burden, defined as an NMSS score of 3, were included in the study (43, 44).

### Sensory behavioral assessment

Mice were habituated to the testing environment for 30 minutes on an elevated wire mesh platform beneath individual transparent Plexiglas chambers in a quiet, temperature-controlled room to minimize stress and exploratory behavior prior to testing. Mechanical sensitivity was assessed using calibrated manual von Frey filaments applied perpendicularly to the plantar surface of both hind paws through the mesh floor. Filaments were presented in ascending order of force according to the up-down method. Each filament was applied five times with sufficient force to produce slight bending, and a response was considered positive when at least three of five applications elicited brisk paw withdrawal, shaking, or licking (45, 46). All testing was conducted by an experimenter blinded to treatment conditions.

### Rotarod test for motor function assessment

Motor coordination and balance were assessed using an accelerating rotarod apparatus (ENV-574, Med Associates, USA). The rotarod was programmed to accelerate linearly from 4 revolutions per minute (rpm) to 40 rpm over a 350-second trial period. Mice were placed individually on the rotating rod, and the latency to fall was recorded automatically by the system. Each mouse completed three consecutive trials, with a 5-minute inter-trial interval to minimize fatigue. The mean latency to fall across the three trials was calculated and used for subsequent statistical analysis (47). All testing was conducted under consistent environmental conditions and within the same time window to reduce variability.

### Electrophysiological assessment

Nerve conduction studies were performed using a PowerLab data acquisition system (AD Instruments, Sydney, Australia) as previously described (48). Mice were anesthetized with 1.5% isoflurane delivered via inhalation and maintained on a thermostatically controlled heating pad throughout the procedure to preserve body temperature. Compound muscle action potentials (CMAPs) were recorded bilaterally from the hind paws using sterile needle electrodes inserted into the plantar muscles of the foot. Electrical stimulation was applied to the sciatic and tibial nerves at the level of the sciatic notch using bipolar stimulating electrodes. CMAP amplitude was defined as the peak-to-peak voltage of the evoked response. Latency was measured as the time from stimulus onset to the initial deflection of the CMAP, and response duration was defined as the interval from response onset to return to baseline.

### Flow cytometric analysis

Peripheral blood, bilaterally pooled sciatic and tibial nerves, and spleen samples were analyzed using a CytoFLEX A flow cytometer (Beckman Coulter, USA). For peripheral blood collection, mice were briefly anesthetized with isoflurane, and approximately 100 µL of blood was obtained from the facial vein into EDTA-coated tubes. Whole blood samples were incubated with fluorochrome-conjugated surface antibody master mixes, followed by erythrocyte lysis using ammonium chloride buffer. Remaining leukocytes were pelleted, washed, and resuspended in cell staining buffer (CSB) prior to data acquisition.

For spleen and nerve tissue processing, mice were perfused with ice-cold PBS to minimize intravascular contamination. Spleens were harvested, mechanically dissociated, subjected to red blood cell lysis, filtered through 70-µm strainers, counted, and stained. Distal 1.5-cm sciatic nerve segments and entire tibial nerves were collected and bilaterally pooled per mouse, then dissociated using combined mechanical disruption and enzymatic digestion, followed by filtration through 70-µm strainers prior to staining, as previously described (44, 49, 50). For all tissues, Zombie Aqua fixable viability dye and TruStain FcX were applied before extracellular antibody staining. For intracellular FcγRIIB/CD32B staining, cells were fixed, permeabilized, and stained according to established protocols (51). Gating strategies for leukocyte and myeloid populations in blood, nerve, and spleen samples are provided in the Supplementary Materials (**Supplementary Figures 1**, **2**), and detailed antibody information (clone, fluorochrome, catalog number, and dilution) is listed in **Table 1**. Data were analyzed using FlowJo software (v10.10.0). 

**Table 1:** Antibodies and reagents used in flow cytometry.

Antibody name	Clone	Supplier	Catalog #	Dilution
TruStain FcX anti-mouse Fc Block	93	Biolegend	101320	1:50
Zombie Aqua Live/Dead	Dye	Biolegend	77143	1:1000
Pacific Blue anti-mouse CD11c	N418	Biolegend	117322	1:50
FcγRIIB (CD32B) XP Rabbit mAb	D8F9C	Cell signaling	54837S	1:50
PE anti-mouse F4/80	W200065D	Biolegend	111704	1:50
PE/Cyanine7 anti-mouse Ly-6C	HK 1.4	Biolegend	128018	1:50
APC anti-mouse/human CD11b	M1/70	Biolegend	101212	1:50
AF700 anti-mouse CD45	I3/2.3	Biolegend	147716	1:50
BV605 anti-mouse CD25	PC61	Biolegend	102036	1:50
PE/Cyanine5 anti-mouse CD3	17A2	Biolegend	100274	1:50
PE anti-mouse CD39	Duha59	Biolegend	143804	1:50
PE/Cyanine7 anti-mouse CD4	RM4-5	Biolegend	100528	1:50
BV605 anti-mouse CD19	6D5	Biolegend	115539	1:50

### Immunohistochemical analysis

Mice were perfused transcardially with PBS followed by 4% formaldehyde. Sciatic nerves were harvested, post-fixed overnight, cryoprotected in sucrose, embedded in optimal cutting temperature (OCT) compound, and sectioned at 10 µm. After blocking, tissue sections were incubated overnight at 4 °C with primary antibodies against CD68 (Bio-Rad, USA), followed by incubation with appropriate fluorescent secondary antibodies. CD68-positive cells were visualized and quantified using a fluorescence microscope (Olympus IX83, Tokyo, Japan) as previously described (35, 52).

### Morphometric analysis of peripheral nerves

Mice were anesthetized with isoflurane and transcardially perfused with ice-cold PBS followed by 4% paraformaldehyde. Sciatic and tibial nerves were dissected and post-fixed overnight at 4 °C in the dark in 3% glutaraldehyde. Toluidine blue staining was performed as previously described (53, 54). Briefly, following glutaraldehyde fixation, nerve samples were post-fixed with 2% osmium tetroxide, dehydrated through graded alcohols, and embedded in epoxy resin. Semi-thin cross-sections (1–2 µm) were cut using an ultramicrotome, mounted on glass slides, and stained with 1% toluidine blue for 20–30 s. Sections were rinsed with deionized water, air-dried, and coverslipped for light microscopic analysis using an Olympus BX51 microscope. All myelinated axons within whole sciatic nerve cross-sections were counted. Demyelination was quantitatively assessed by calculating the g-ratio, defined as the ratio of the inner axonal diameter to the total outer fiber diameter (axon + myelin). Digital images were captured at high magnification, and measurements were obtained using ImageJ software.

### Statistical analysis

Phenotypic outcomes were analyzed using two-way repeated-measures analysis of variance (ANOVA) followed by Tukey’s multiple-comparisons *post hoc* test when appropriate. Where applicable, two-group comparisons were performed using Student’s t-test. Flow cytometry endpoints and histological measurements were compared across groups using one-way ANOVA with Tukey’s *post hoc* test. Data normality was assessed using the Shapiro–Wilk test prior to parametric analyses. All data are presented as mean ± SEM.

## Results

### NVG-2089 provides neuroprotection comparable to high-dose IVIg in experimental demyelinating neuropathy

SAPP is a well-established, T-cell–orchestrated murine model of inflammatory demyelinating peripheral neuropathy that recapitulates key immunopathological and clinical features of human immune-mediated neuropathies (37, 42). Disease penetrance in this model is strongly sex-biased, exceeding 90% in females but occurring in only ~30% of males (37); therefore, female mice were used exclusively for all experiments to ensure robust and reproducible disease induction. We have previously characterized the temporal clinical progression, histopathological changes, and immune mechanisms underlying neuropathy in SAPP mice, and have used this platform to evaluate immunoprotective therapeutic strategies (35).

To directly compare the efficacy of NVG-2089 with high-dose IVIg, disease progression was monitored longitudinally over a 28-day period using a comprehensive battery of behavioral and functional assessments. These included the NMSS to evaluate overall motor impairment, von Frey filament testing to assess sensory function and mechanical sensitivity, and rotarod performance to measure motor coordination and balance.

In vehicle-treated control animals, NMSS increased steadily over time, reflecting progressive neuromuscular deterioration and worsening motor deficits (**Figure 1B**). In contrast, treatment with either NVG-2089 (****p < 0.0001) or IVIg (***p = 0.0004) effectively halted disease progression, with scores stabilizing shortly after treatment initiation. Notably, this protective effect was sustained through the end of the observation period at day 26, indicating durable functional benefit. Mechanical sensitivity assessments revealed a similar pattern. Control mice exhibited progressive increases in von Frey withdrawal thresholds beginning around day 8, consistent with advancing sensory dysfunction (**Figure 1C**). By comparison, animals treated with NVG-2089 or IVIg maintained withdrawal thresholds near baseline values or showed only modest decreases over time. These differences were highly significant (****p < 0.0001), demonstrating preservation of sensory function with both treatments. Motor coordination and balance, as assessed by rotarod testing, declined progressively in control mice, with a marked reduction in latency to fall evident from day 5 onward (**Figure 1D**). In contrast, both NVG-2089- and IVIg-treated groups maintained near-baseline rotarod performance across multiple testing time points. On the final day of the study, treated animals remained on the rod significantly longer than controls, with mean latencies of 124.9 ± 31.0 s for NVG-2089 and 130.0 ± 45.1 s for IVIg, compared with only 9.66 ± 2.5 s in control mice (*p < 0.05).

**Figure 2:**
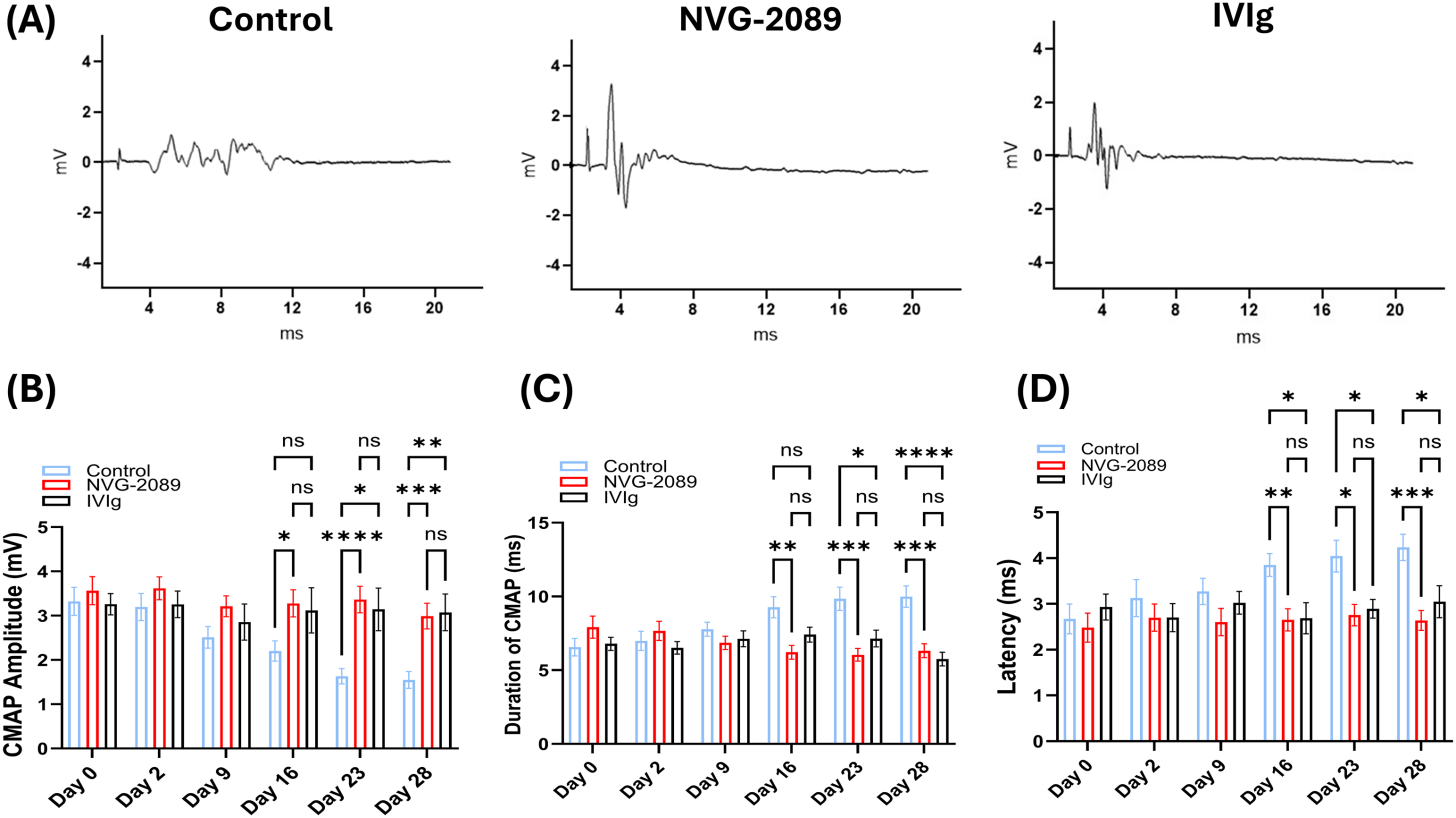
Electrophysiological outcomes following NVG-2089 or IVIg treatment. **(A)** Representative compound muscle action potential (CMAP) traces from control, NVG-2089 treated, and IVIg-treated animals. **(B)** Longitudinal analysis of CMAP amplitude demonstrated significant preservation in NVG-2089– and IVIg-treated groups compared with controls at later time points, consistent with maintained axonal integrity. **(C)** CMAP duration progressively increased in control animals, indicative of demyelination and temporal dispersion, whereas NVG-2089 and IVIg limited this prolongation. **(D)** CMAP latency increased over time in control animals but remained significantly lower in NVG-2089– and IVIg-treated groups, reflecting improved conduction properties. Data are presented as mean ± SEM with individual data points shown. *p < 0.05, **p < 0.01, ***p < 0.001, ****p < 0.0001; ns, not significant.

Electrophysiological findings closely paralleled these behavioral outcomes. Representative CMAP traces obtained at the study endpoint illustrate the severely attenuated responses in control animals compared with the preserved waveform amplitudes observed following NVG-2089 or IVIg treatment (**Figure 2A**). In control mice, CMAP amplitudes began to decline by day 16 and decreased further by day 28, reaching 1.54 ± 0.18 mV, consistent with progressive axonal dysfunction and impaired neuromuscular transmission. In contrast, both NVG-2089 and IVIg maintained stable CMAP amplitudes throughout the study, with no significant difference between treatments at day 28 (2.99 ± 0.28 mV and 3.07 ± 0.41 mV, respectively) (**Figure 2B**).

Similarly, CMAP duration also broadened progressively in controls between days 16 and 28 (day 28: 9.99 ± 0.72 ms), whereas durations remained significantly shorter in both treatment groups, measuring 6.32 ± 0.47 ms with NVG-2089 (***p = 0.0006) and 5.76 ± 0.46 ms with IVIg (****p < 0.0001) (**Figure 2C**). CMAP conduction latency was significantly prolonged in control animals from day 16 onward, reflecting demyelination and slowed nerve conduction (**Figure 2D**). Both treatments effectively prevented this latency prolongation. At day 28, CMAP latency was 4.24 ± 0.28 ms in controls, compared with 2.64 ± 0.21 ms in NVG-2089–treated mice (***p = 0.0003) and 3.05 ± 0.35 ms in IVIg-treated mice (*p = 0.037 vs. control).

Taken together, these behavioral and electrophysiological data demonstrate that NVG-2089 provides robust and sustained protection against motor dysfunction, sensory impairment, and nerve conduction abnormalities in the SAPP model. Across all measured endpoints, NVG-2089 matched the efficacy of high-dose IVIg, supporting its potential as a therapeutically equivalent alternative in immune-mediated demyelinating neuropathy.

### NVG-2089 and IVIg preserve myelin architecture and reduce macrophage infiltration

**Figure 3:**
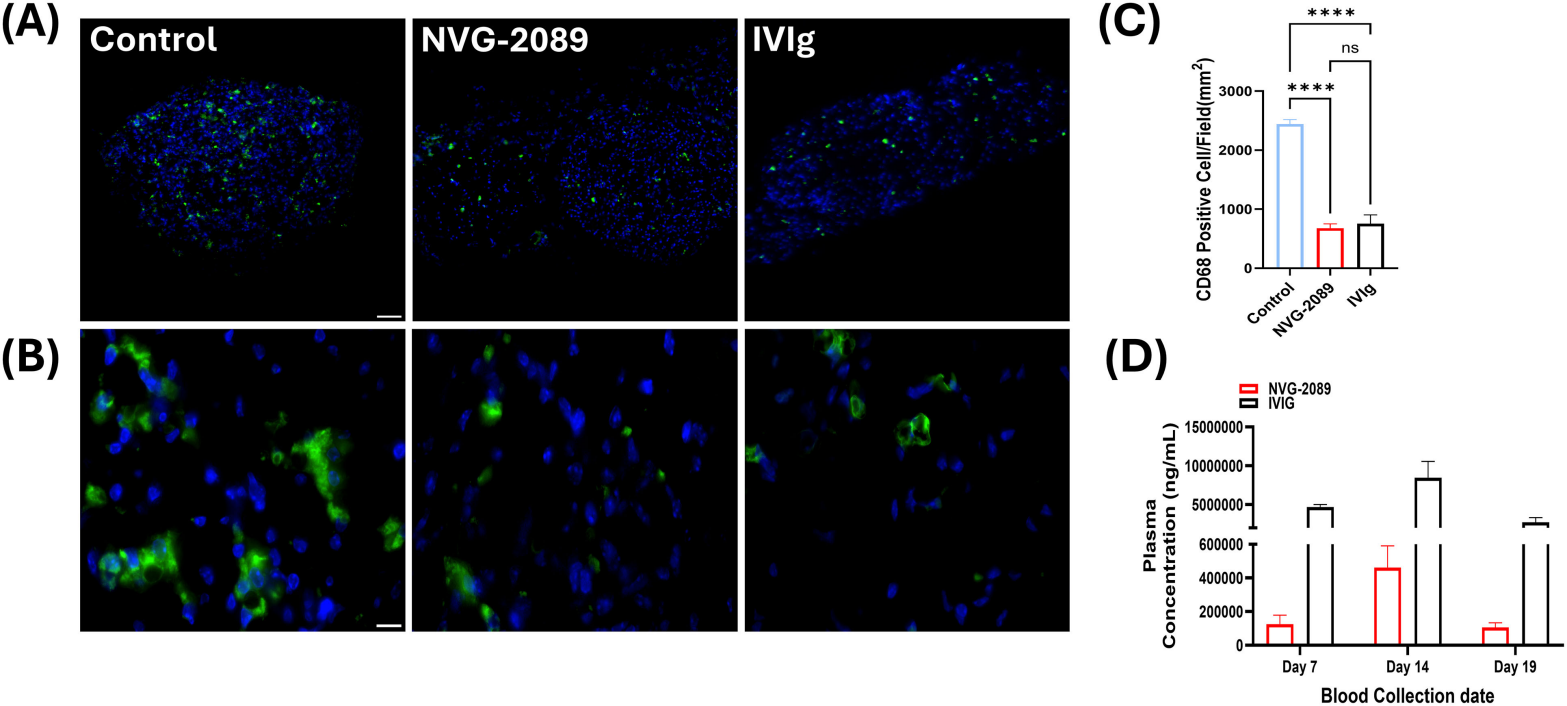
NVG-2089 and IVIg attenuate macrophage accumulation in sciatic nerves of symptomatic SAPP mice. **(A, B)** Representative immunofluorescence images demonstrating CD68^+^ macrophages (green) with DAPI nuclear counterstain (blue) in control, NVG-2089–treated, and IVIg-treated groups [**(A)** 20×; **(B)** 60× magnification]. **(C)** Quantification of CD68^+^ cell density (cells/mm²) revealed significant macrophage accumulation in control nerves, whereas both NVG-2089 and IVIg markedly reduced inflammatory cell infiltration. No significant difference was observed between the two treatment groups. **(D)** Plasma concentrations of NVG-2089 and IVIg measured on days 7, 14, and 19 following initial treatment confirmed sustained systemic exposure for both agents. Scale bars: 50 µm **(A)** and 10 µm **(B)**. Data are presented as mean ± SEM with individual data points shown. ****p < 0.0001; ns, not significant.

Immunohistochemical analysis revealed consistent reductions in peripheral nerve inflammation following treatment with NVG-2089 or IVIg. Representative low-magnification (20X) images of sciatic nerve cross sections demonstrated dense accumulation of CD68^+^ macrophages throughout the endoneurium of PBS-treated SAPP mice. In contrast, nerves from NVG-2089- and IVIg-treated animals exhibited markedly fewer CD68^+^ cells with a more diffuse distribution (**Figure 3A**). These qualitative differences were further evident at higher magnification (60X), where PBS-treated nerves displayed prominent clusters of CD68^+^ macrophages, whereas NVG-2089- and IVIg-treated sections showed sparse CD68^+^ signal with minimal cellular aggregation (**Figure 3B**).

Quantitative analysis confirmed these observations, showing significantly elevated CD68^+^ cell density in controls (2444 ± 75 cells/mm²), which was robustly reduced (****p < 0.0001 for each) by both NVG-2089 (676 ± 72.1 cells/mm², ****p < 0.0001 vs control) and IVIg (752.8 ± 151 cells/mm², ****p < 0.0001 vs control). No significant difference was observed between the two treatment groups (**Figure 3C**). This reduction in macrophage burden is consistent with the preservation of electrophysiological function and myelin integrity observed in treated animals.

To confirm sustained systemic exposure during the treatment period, circulating levels of NVG-2089 and IVIg were measured in serial serum samples collected on days 7, 14, and 19 following the initial treatment (**Figure 3D**). Although NVG-2089 was administered at a tenfold lower dose than IVIg (100 mg/kg vs. 1 g/kg), both agents exhibited measurable serum concentrations across all time points, supporting ongoing systemic availability. Notably, NVG-2089 has a shorter serum half-life than IVIg (21), which would typically be expected to result in more rapid systemic clearance and reduced persistence in circulation. Despite this, NVG-2089 remained detectable at all sampled time points, suggesting sustained systemic exposure and supporting its pharmacokinetic robustness even at a lower dose. Importantly, these exposure profiles were associated with comparable biological efficacy, as both treatments significantly reduced CD68^+^ macrophage infiltration relative to controls, with no significant difference observed between treatment groups (**Figure 3C**).

**Figure 4:**
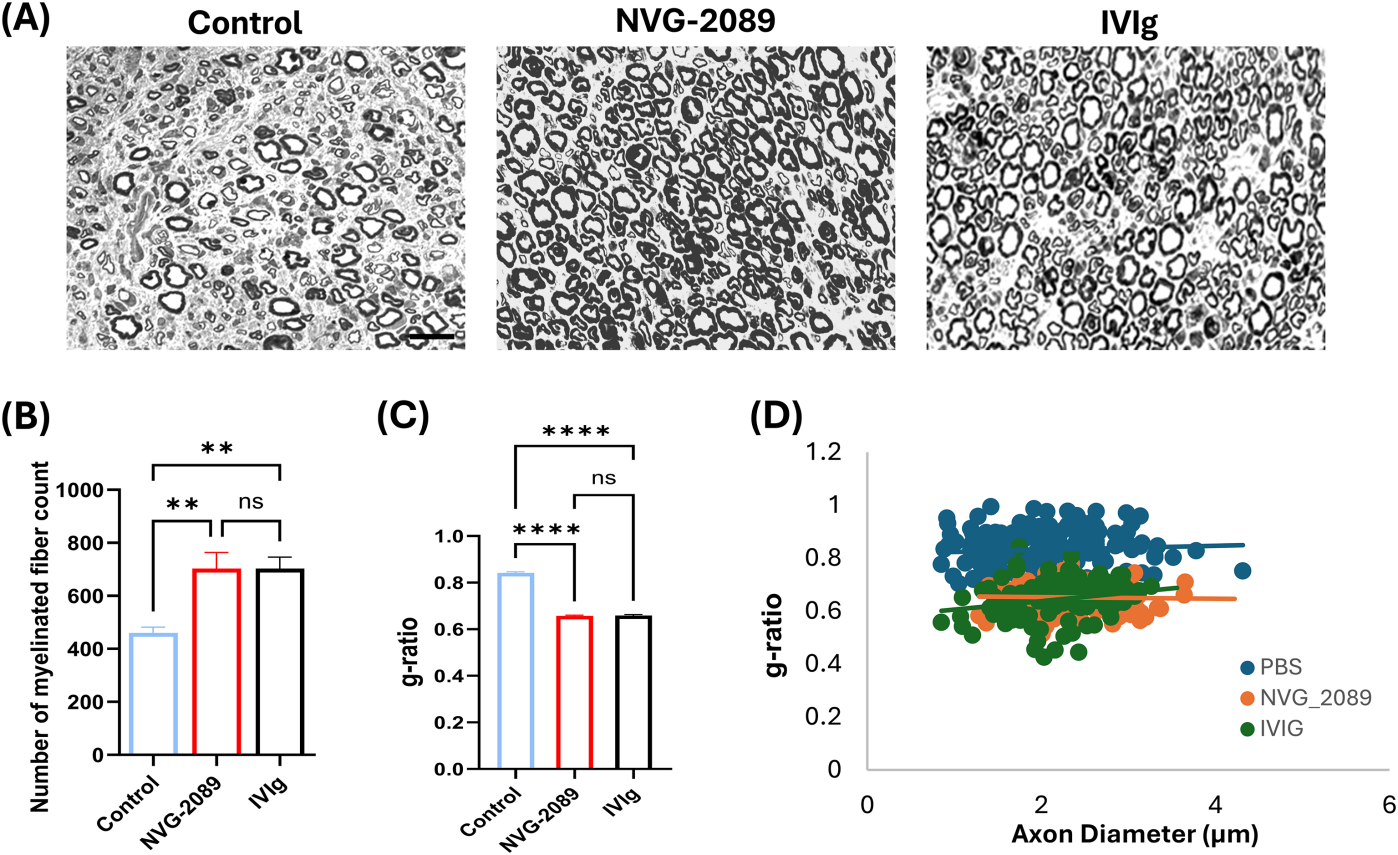
NVG-2089 and IVIg preserve myelin architecture and increase myelinated fiber number. **(A)** Representative semi-thin cross-sections of sciatic nerve from control, NVG-2089 treated, and IVIg-treated groups illustrating overall myelin integrity and axonal preservation (Scale bar: 20 µm). **(B)** Myelinated fiber counts increased significantly with NVG-2089 or IVIg treatment compared with controls, with no detectable difference between treatments. **(C)** Group comparison of mean g-ratio demonstrated significantly lower values in NVG-2089 and IVIg-treated animals compared with controls, consistent with relatively thicker myelin. No significant difference was observed between the two treatment groups. **(D)** Scatter plot of g-ratio versus axon diameter for individual myelinated fibers across groups. Data are presented as mean ± SEM with individual data points shown. **p < 0.01, ****p < 0.0001; ns, not significant.

Morphometric analysis of toluidine blue–stained sciatic nerve cross sections demonstrated significant preservation of myelinated axons following treatment (**Figure 4A**). Myelinated fiber counts were significantly reduced in control mice (460 ± 22) compared with animals treated with NVG-2089 (702.5 ± 61.4, **p = 0.0048 vs. control) or IVIg (702.7 ± 43.4; **p = 0.0048 vs. control), with no difference between the two treatment groups (**Figure 4B**). Consistent with these findings, g-ratio analysis revealed marked myelin thinning in PBS-treated nerves (0.841 ± 0.004), which was significantly improved by both NVG-2089 (0.6586 ± 0.003) and IVIg (0.6584 ± 0.0044; ****p < 0.0001 vs. control), again without differences between two treatments (**Figures 4C, D**).

### NVG-2089 and IVIg increase regulatory T-cell frequency and enhance FcγRIIB expression

To investigate immunological mechanisms underlying the neuroprotective effects of NVG-2089 and IVIg in inflammatory demyelinating neuropathy, flow cytometry was performed across systemic and peripheral nerve tissues using a prespecified gating strategy (**Supplementary Figures 1**, **2**). Tissue-specific populations were defined as follows: regulatory T cells (Tregs) were identified as CD3^+^CD4^+^CD25^+^CD39^+^ cells; B cells as CD45^+^CD11b^-^CD19^+^; monocytes as CD45^+^CD11b^+^Ly6C^+^; endoneurial dendritic cells (DCs) as CD45^+^CD11b^+^CD11c^+^F4/80^-^; and macrophages as CD45^+^CD11b^+^CD11c^+^F4/80^+^. Positive gates were established using fluorescence minus one (FMO) controls, and expression of the inhibitory Fc receptor FcγRIIB (CD32B) was quantified both as the frequency of FcγRIIB^+^ cells and by mean fluorescence intensity (MFI).

**Figure 5:**
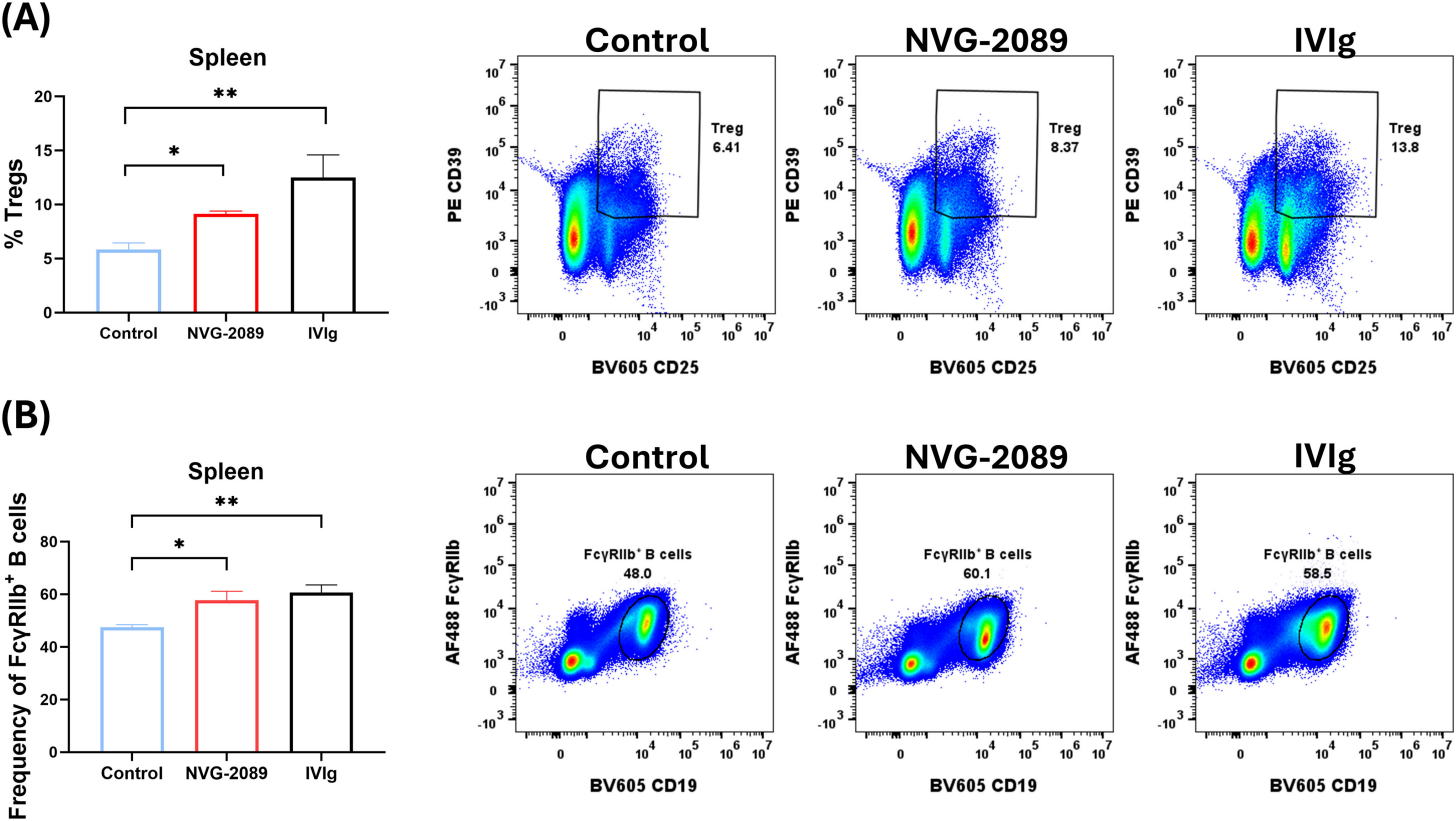
NVG-2089 and IVIg expand splenic regulatory T cells and FcγRIIB^+^ B-cell populations. **(A)** Flow cytometric quantification of splenic regulatory T cells (Tregs). Bar graph shows the percentage of Tregs among total CD4^+^ T cells in control, NVG-2089–treated, and IVIg-treated groups. **(B)** Flow cytometric analysis of splenic FcγRIIB^+^ B cells. Bar graph shows the frequency of FcγRIIB^+^ B cells across groups. Both NVG-2089 and IVIg increased the frequency of immunoregulatory Tregs and inhibitory FcγRIIB^+^ B cells compared with controls, consistent with enhanced peripheral immune regulation. Data are presented as mean ± SEM with individual data points shown. n=8 mice per group. *p < 0.05, **p < 0.01; ns, not significant.

In the spleen, both NVG-2089 and IVIg significantly increased the frequency of CD4^+^CD25^+^CD39^+^ Tregs compared with controls, with Treg frequencies of 9.13 ± 0.27% and 12.5 ± 2.1%, respectively (**Figure 5A**). This expansion of the regulatory compartment suggests enhanced immune restraint at the systemic level. In parallel, both treatments increased the proportion of FcγRIIB-expressing splenic B cells relative to controls (**Figure 5B**), consistent with a shift toward inhibitory Fc receptor signaling in humoral immune cells.

**Figure 6:**
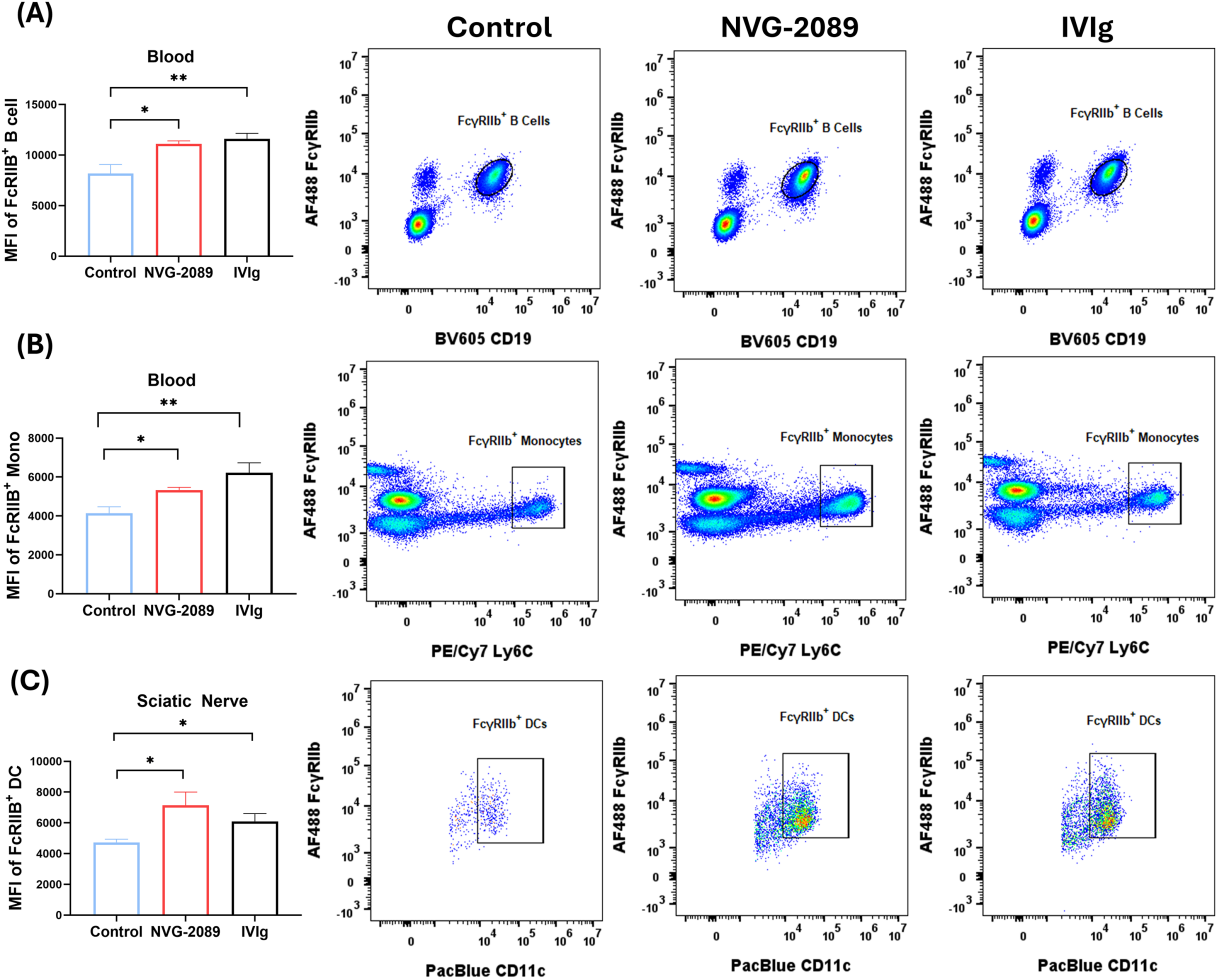
NVG-2089 and IVIg enhance FcγRIIB expression across circulating and nerve-resident immune cell subsets. **(A-C)** FcγRIIB mean fluorescence intensity (MFI) was quantified by flow cytometry on **(A)** circulating B cells (FcγRIIB^+^CD19^+^), **(B)** circulating monocytes (FcγRIIB^+^Ly6C^+^), and **(C)** dendritic cells (FcγRIIB^+^ DCs) within peripheral nerve tissue. Both NVG-2089 and IVIg significantly increased FcγRIIB expression across multiple immune cell populations compared with controls. Data are presented as mean ± SEM with individual data points shown. *p < 0.05, **p < 0.01.

Comparable immunomodulatory effects were observed in the peripheral circulation. In peripheral blood, FcγRIIB expression on B cells was significantly elevated in both NVG-2089- and IVIg-treated mice compared with controls (**Figure 6A**). Similarly, circulating monocytes exhibited significantly elevated FcγRIIB expression following treatment with either IVIg or NVG-2089 (**Figure 6B**). These findings indicate that both agents enhance FcγRIIB expression across key adaptive and innate immune cell populations in the periphery, supporting a systemic immunomodulatory mechanism of action.

Importantly, FcγRIIB upregulation was also evident within the target tissue. Myeloid DCs isolated from sciatic nerves of NVG-2089 and IVIg-treated mice exhibited significantly higher FcγRIIB expression than controls (**Figure 6C**), indicating local engagement of inhibitory FcγRIIB-associated pathways at the site of nerve injury. This local increase in FcγRIIB expression is consistent with reduced macrophage infiltration and preservation of myelin architecture observed histologically.

Collectively, these data demonstrate that NVG-2089, similar to high-dose IVIg, is associated with increased regulatory T cells and enhanced FcγRIIB expression across systemic and peripheral nerve immune compartments. The concordant upregulation of these immunoregulatory features in spleen, blood, and sciatic nerve suggests engagement of shared inhibitory immune pathways by both agents. These immune changes occur in parallel with reduced macrophage infiltration, preserved myelin architecture, and improved electrophysiological and behavioral outcomes in the SAPP model.

## Discussion

IVIg remains an effective therapy for both acute and chronic inflammatory demyelinating neuropathies (55–57). However, its widespread clinical use is limited by high-dose requirements, substantial cost, finite global supply, and the logistical burden of repeated infusions (58). These challenges have become increasingly relevant as demand for IVIg continues to rise across multiple neurological and immunological indications. There is therefore a growing need for next-generation immunomodulatory therapies that retain the therapeutic benefits of IVIg while improving dose efficiency, scalability, and accessibility. In this study, we demonstrate that NVG-2089, a recombinant IgG1 Fc engineered to recapitulate key immunoregulatory properties of sialylated IgG (21), achieves robust neuroprotection in a preclinical inflammatory demyelinating neuropathy model at one-tenth the dose of IVIg.

NVG-2089 produced therapeutic effects that closely mirrored those of high-dose IVIg across multiple clinically relevant endpoints, including preservation of motor and sensory function, stabilization of electrophysiological parameters, attenuation of inflammatory cell infiltration within peripheral nerves, and maintenance of myelin architecture. The consistency of these effects across functional, structural, and immunological domains underscores the translational potential of NVG-2089 as a dose-efficient alternative to IVIg rather than a narrowly acting experimental agent.

IVIg is known to exert its therapeutic effects through multiple mechanisms, including increasing the activation threshold of innate effector cells to immune complexes by induction of FcγRIIB, modulation of complement activity, and expansion of regulatory T cells, as demonstrated in both clinical and experimental settings (54, 59–64). Consistent with these established mechanisms, our study demonstrated that treatment with either NVG-2089 or IVIg was associated with increased frequencies of regulatory T cells and enhanced expression of the inhibitory Fc receptor FcγRIIB across systemic and peripheral nerve immune compartments. Notably, these immunomodulatory changes were detected not only in lymphoid tissues and circulation but also locally within the sciatic nerve, indicating that NVG-2089 affects regulatory pathways directly at sites of tissue injury.

Previous studies have demonstrated that the anti-inflammatory activity of IVIg and sialylated IgG Fc fragments *in vivo* requires engagement of the lectin receptor SIGN-R1 in mice, or its human ortholog DC-SIGN (65). SIGN-R1 signaling has been linked to downstream induction of IL-33, which promotes expansion of IL-4–producing basophils and subsequent upregulation of the inhibitory Fc receptor FcγRIIB on effector macrophages (62). Importantly, the anti-inflammatory activity of NVG-2089 is likewise dependent on SIGN-R1 (21), suggesting that this upstream lectin pathway may represent a shared initiating signal for both agents. Within this framework, the increased FcγRIIB expression observed across immune subsets in our study is consistent with activation of a SIGN-R1–IL-33–basophil axis that ultimately enhances inhibitory Fc signaling. Although the present data do not directly examine this cascade, the parallel immunophenotypic changes support mechanistic consistency with established IVIg and sialylated Fc biology.

Treg deficiency or dysfunction has been reported during the acute phase of GBS, as well as during the progressive or relapse phases of CIDP, with reduced frequencies of CD4^+^CD25^+^ T cells correlating with disease severity (66–68). IVIg has been shown to restore this imbalance through mechanisms involving cyclooxygenase-2–dependent prostaglandin E_2_ production in dendritic cells (69, 70). Evidence from additional autoimmune models further supports this interpretation. NVG-2089 and sialylated Fc protect mice in the antibody-mediated K/BxN arthritis model and suppress disease in the T cell–mediated experimental autoimmune encephalomyelitis model, where therapeutic benefit has been linked to activation and expansion of regulatory T cells (31). In both antibody- and T cell-driven settings, protection by these anti-inflammatory Fc fragments requires SIGN-R1 signaling and IL-33 induction, underscoring the importance of this lectin-dependent regulatory pathway. Consistent with these observations, our study demonstrated that both NVG-2089 and IVIg significantly increased splenic CD4^+^CD25^+^CD39^+^ Tregs, indicating expansion of a highly suppressive regulatory subset and suggesting enhanced systemic immune regulation. CD39 marks functionally potent Tregs (71–73) that produce IL-10 and mediate suppression via adenosine generation with CD73, limiting Th1 and Th17 responses and effector T cell activation (74, 75). The replication of similar immunoregulatory signatures in the present neuropathy model suggests that NVG-2089 engages a conserved Fc-dependent inhibitory program across distinct autoimmune contexts.

The regulatory role of FcγRIIB across immune cell subsets further supports its relevance as a central mechanism of action. In B cells, FcγRIIB functions as a critical negative regulator of B-cell receptor signaling, suppressing antibody production and limiting autoantibody-mediated tissue injury (33). The increased frequency of FcγRIIB-expressing B cells observed in the spleen and peripheral blood following NVG-2089 treatment may therefore contribute to attenuation of pathogenic humoral responses, a mechanism previously attributed to IVIg (76). In monocytes and other myeloid cells, FcγRIIB counterbalances activating Fcγ receptor ITAM signaling, thereby restraining phagocytosis, reactive oxygen species generation, and pro-inflammatory cytokine release (34, 36). In line with this inhibitory role, both NVG-2089 and IVIg treatment were associated with reduced accumulation of CD68^+^ macrophages within sciatic nerves, reflecting diminished local inflammatory burden and preservation of myelin integrity.

Together, the therapeutic similarity of NVG-2089 and IVIg observed in this study highlights the central importance of FcγRIIB-mediated inhibitory signaling in the control of neuroinflammation. Restoration or amplification of this inhibitory axis may represent a key mechanism by which NVG-2089 achieves efficacy comparable to IVIg in inflammatory demyelinating neuropathies. These findings, together with evidence from antibody- and T cell–mediated autoimmune models (31, 54, 76), support continued development of FcγRIIB-targeted, dose-efficient biologics such as NVG-2089 as next-generation immunomodulators.

The dose efficiency demonstrated by NVG-2089 has important implications for clinical translation. Compared with plasma-derived IVIg, recombinant Fc-based biologics offer advantages in manufacturing consistency, supply stability, and the potential for cost reduction. A therapy capable of achieving IVIg-like efficacy at substantially lower doses could reduce treatment burden, expand patient access, and enable more flexible dosing strategies, particularly in chronic treatment settings. The present data provide preclinical proof-of-concept that FcγRIIB-targeted immunomodulation can deliver meaningful neuroprotection without reliance on gram-scale immunoglobulin dosing.

Several limitations should be considered when interpreting these findings. First, although the SAPP model recapitulates key immunopathological and functional features of inflammatory demyelinating neuropathies, it does not fully capture the clinical and immunological heterogeneity observed in patients with GBS or CIDP. In addition, all SAPP studies were conducted in female mice because disease penetrance in this model is markedly higher in females (>90%) than in males (~30%), enabling consistent disease induction and adequate statistical power. While this sex selection is justified by the biology of the model, it may limit the generalizability of the findings to males. Future studies should therefore evaluate NVG-2089 in both female and male SAPP mice and in complementary models, including antibody-mediated and chronic relapsing disease paradigms (77–79), to determine whether its therapeutic effects extend across sex and distinct forms of immune-mediated neuropathy and to better define its therapeutic breadth. Second, while the observed associations between NVG-2089 treatment, regulatory T-cell expansion, and FcγRIIB upregulation are compelling, the present study does not establish direct causality. Genetic or pharmacologic disruption of FcγRIIB signaling, as well as cell type–specific depletion studies, will be necessary to delineate the relative contributions of individual immune compartments to the observed neuroprotection. Third, this study focused on short- to intermediate-term outcomes; longer-term dosing and recovery studies will be required to assess durability of benefit, potential immunosuppressive liabilities, and relevance to chronic treatment paradigms such as CIDP. Finally, although NVG-2089 demonstrated marked dose efficiency relative to IVIg, formal pharmacokinetic–pharmacodynamic modeling and head-to-head efficacy studies in clinically relevant settings will be essential to inform optimal dosing strategies and guide translation into human trials.

In conclusion, our study demonstrates that NVG-2089 confers robust neuroprotective and immunomodulatory effects comparable to high-dose IVIg, despite being administered at one-tenth of the dose. The associated expansion of regulatory T cells and enhancement of FcγRIIB expression across systemic and peripheral nerve immune compartments provide mechanistic support for its therapeutic activity. Collectively, these findings position NVG-2089 as a promising, dose-efficient, and potentially more accessible alternative to IVIg for the treatment of immune-mediated demyelinating neuropathies, including both GBS and CIDP, and warrant further investigation in translational and clinical settings.

## Data Availability

The original contributions presented in the study are included in the article/**Supplementary Material**. Further inquiries can be directed to the corresponding authors.

## Supplementary material

Supplementary Figure 1 Flow cytometry gating strategy for FcγRIIB expression on circulating monocytes and B cells. Representative gating workflow used to quantify FcγRIIB (AF488) expression on monocytes and B cells in peripheral blood. Events were initially gated by forward and side scatter (FSC/SSC) to exclude debris, followed by singlet discrimination (FSC-A vs. FSC-H) and selection of live cells (Zombie Aqua–negative). Leukocytes were identified as CD45⁺ cells. Myeloid cells were gated as CD45⁺CD11b⁺ cells, and monocytes were further defined as CD11b⁺Ly6C⁺ cells. FcγRIIB-positive monocytes were quantified as Ly6C⁺FcγRIIB⁺ events. Lymphocytes were gated as CD45⁺CD11b⁻ cells, and B cells were identified as CD19⁺ lymphocytes. FcγRIIB-positive B cells were quantified as CD19⁺FcγRIIB⁺ events.

Supplementary Figure 2 Flow cytometry gating strategy for myeloid and T-cell populations in spleen and sciatic nerve. Representative gating workflow for analysis of myeloid and T-cell subsets in spleen and sciatic nerve. For the myeloid panel, leukocytes were first gated as CD45⁺ cells and subsequently subdivided based on CD11b expression into myeloid (CD11b⁺) and lymphoid (CD11b⁻) populations. Within the CD11b⁺ compartment, macrophages were identified as F4/80⁺ cells, and dendritic cells (DCs) were defined as F4/80⁻CD11c⁺ cells. FcγRIIB-positive myeloid were quantified as F4/80⁺FcγRIIB⁺ macrophages and CD11c⁺FcγRIIB⁺ DCs. For the T-cell panel, splenic T lymphocytes were gated as CD3⁺ cells. CD4⁺ T helper cells were identified as CD3⁺CD4⁺ cells. Regulatory T cells (Tregs) were defined within the CD4⁺ T-cell population as CD25⁺CD39⁺ cells.
