# Supplementary figures and images for: A recombinant IgG1 Fc-domain protein ameliorates inflammatory demyelinating peripheral neuropathy
Source: Front Immunol. 2026 May 27;17:1857016. doi: 10.3389/fimmu.2026.1857016 (PMC13251368; doi:10.3389/fimmu.2026.1857016)

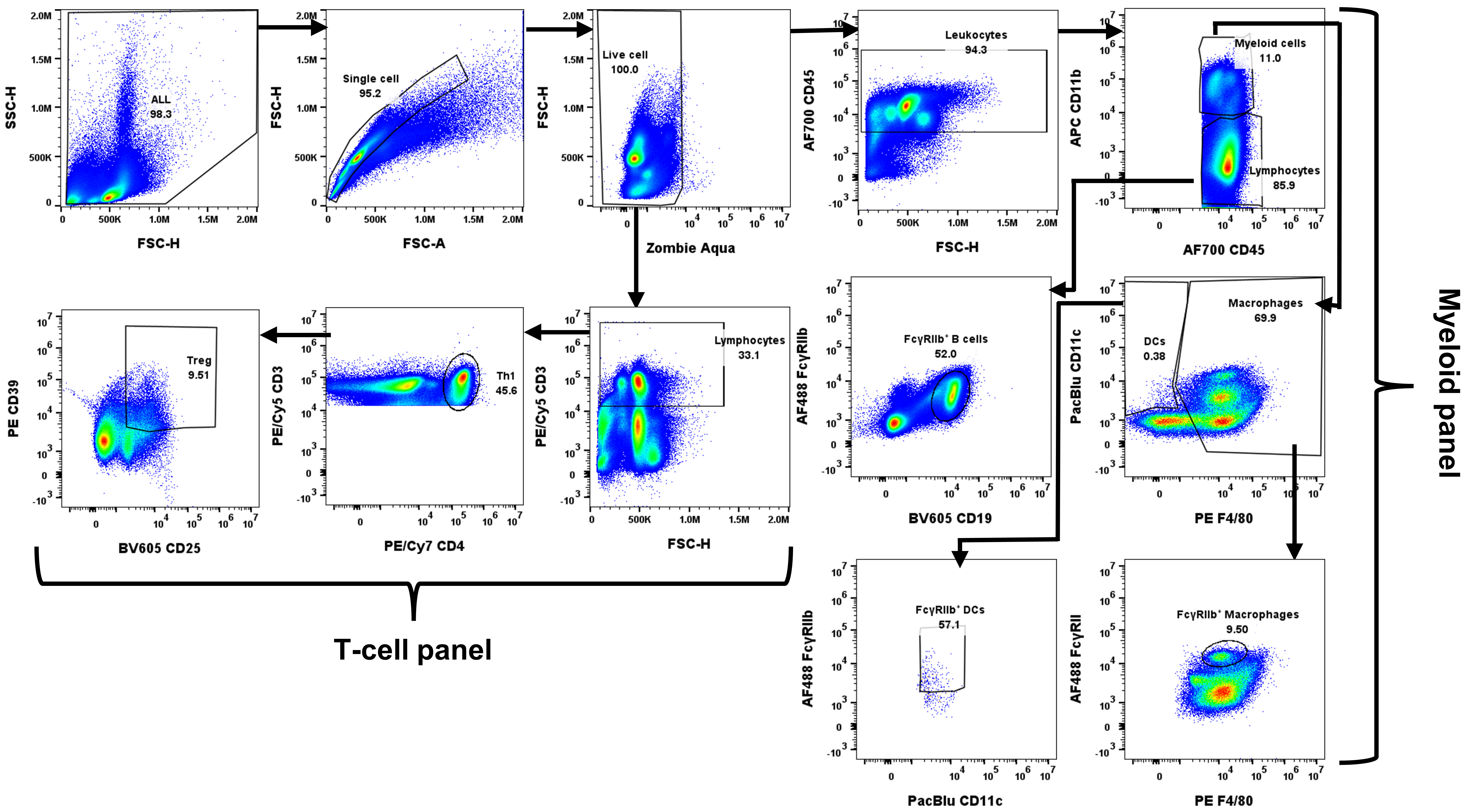

Supplement: Supplementary Figure 2 — Flow cytometry gating strategy for myeloid and T-cell populations in spleen and sciatic nerve. Representative gating workflow for analysis of myeloid and T-cell subsets in spleen and sciatic nerve. For the myeloid panel, leukocytes were first gated as CD45⁺ cells and subsequently subdivided based on CD11b expression into myeloid (CD11b⁺) and lymphoid (CD11b⁻) populations. Within the CD11b⁺ compartment, macrophages were identified as F4/80⁺ cells, and dendritic cells (DCs) were defined as F4/80⁻CD11c⁺ cells. FcγRIIB-positive myeloid were quantified as F4/80⁺FcγRIIB⁺ macrophages and CD11c⁺FcγRIIB⁺ DCs. For the T-cell panel, splenic T lymphocytes were gated as CD3⁺ cells. CD4⁺ T helper cells were identified as CD3⁺CD4⁺ cells. Regulatory T cells (Tregs) were defined within the CD4⁺ T-cell population as CD25⁺CD39⁺ cells. [file Image2.tif]
